# MiR-124 Radiosensitizes Human Colorectal Cancer Cells by Targeting PRRX1

**DOI:** 10.1371/journal.pone.0093917

**Published:** 2014-04-04

**Authors:** Yuqin Zhang, Lin Zheng, Jing Huang, Fei Gao, Xiaoshan Lin, Lian He, Dan Li, Zhijun Li, Yi Ding, Longhua Chen

**Affiliations:** 1 Department of Radiation Oncology, Nanfang Hospital, Southern Medical University, Guangzhou, Guangdong, China; 2 Department of Pathology, School of Basic Medical Sciences, Southern Medical University, Guangzhou, Guangdong, China; 3 Department of Cancer Center of Affiliated Hospital, Guangdong Medical College, Zhanjiang, Guangdong, China; 4 Department of Gastroenterology, The First Affiliated Hospital of Jinan University, Guangzhou, Guangdong, China; University of Barcelona, Spain

## Abstract

One of the challenges in the treatment of colorectal cancer patients is that these tumors show resistance to radiation. MicroRNAs (miRNAs) are involved in essential biological activities, including chemoresistance and radioresistance. Several research studies have indicated that miRNA played an important role in sensitizing cellular response to ionizing radiation (IR). In this study, we found that miR-124 was significantly down-regulated both in CRC-derived cell lines and clinical CRC samples compared with adjacent non-tumor colorectal tissues, MiR-124 could sensitize human colorectal cancer cells to IR *in vitro* and *in vivo*. We identified PRRX1, a new EMT inducer and stemness regulator as a novel direct target of miR-124 by using target prediction algorithms and luciferase assay. PRRX1 knockdown could sensitize CRC cells to IR similar to the effects caused by miR-124. Overexpression of PRRX1 in stably overexpressed-miR-124 cell lines could rescue the effects of radiosensitivity enhancement brought by miR-124. Taking these observations into consideration, we illustrated that miR-124 could increase the radiosensitivity of CRC cells by blocking the expression of PRRX1, which indicated miR-124 could act as a great therapeutic target for CRC patients.

## Introduction

Colorectal cancer (CRC) is the third leading cause of cancer deaths [1]. CRC cases are treated through surgical resection as it is the only curative technique that is available till date. Nevertheless, colorectal cancer is associated with high mortality as approximately 30% of patients are diagnosed when this cancer has reached an advanced stage. These patients harbor a locally advanced, unresectable, non-metastatic disease that is termed as “locally advanced colorectal cancer.” These patients received chemoradiation therapy. Nevertheless, because of the inherent ability of colorectal cancer to become chemoresistant and radioresistant (RR), combined modality therapy has failed to universally improve outcomes. It is difficult to treat patients with locally advanced colorectal cancer because these cancer cells show resistance to radiation therapy. This is one of the most challenging issues in the treatment of colorectal cancer. Therefore, we need to elucidate the molecular mechanisms underlying radiation sensitivity or resistance as this would ultimately help us in improving therapeutic outcomes.

Previous studies have confirmed an association between radioresistance and the expression of genes that induce the DNA damage checkpoint response and increase DNA repair capacity [2]–[4]. Although such discoveries have improved the understanding of molecular mechanisms that influence cellular radiosensitivity, the detailed mechanisms by which this process is regulated is not known till date.

MicroRNAs (miRNAs) are a class of small (*∼22 nucleotides*) non-coding RNA molecules that regulate post-transcriptional gene expression. By binding with partially complementary sequences of messenger RNA (mRNA), miRNAs target mRNA molecules for degradation and/or inhibit translation, thereby decreasing the expression of proteins [5]. Several experimental evidences have suggested that several human diseases, including cancer have been caused due to the deregulation of miRNAs [6]. Some miRNAs modulate the expression of known oncogenes or tumor suppressor genes, whereas others function as oncomiRNAs or tumor-suppressor miRNAs [7]. Several miRNAs have been correlated with patient survival. These miRNAs may be useful in predicting and modifying anticancer treatments (including chemotherapy, radiotherapy, and immunotherapy)[8], [9]. In recent times, we have discovered that miRNAs played an important role in the cellular response to ionizing radiation [10]–[13].

MiR-124 is a brain-enriched miRNA, which is significantly down-regulated in many human malignant tumors, including glioblastoma, gastric carcinoma, medulloblastoma, hepatocellular carcinoma, and CRC [14]–[22]. Recent studies have shown that miR-124 radiosensitizes human glioma cells by targeting CDK4 [23]. However, the function of miR-124 on radioresistance has been scarcely understood till date.

PRRX1 is a newly identified EMT (the epithelial-mesenchymal transition) inducer and stemness regulator [24], [25], both of which are closely related to radioresistence [26]. In addition, abundant PRRX1 expression was correlated with poor prognosis and metastasis in CRC cases. Moreover, abundant expression of PRRX1 was also associated with poor clinical outcomes [27]. Recent studies have also shown that PRRX1 played a pivotal role in pancreatic regeneration and carcinogenesis [28]. In this study, we illustrated that miR-124 enhanced the sensitivity to radiation, both in CRC cells and human xenograft tumors. Moreover, PRRX1 is a novel, direct target of miR-124 that induces irradiation resistance. PRRX1 knockdown could sensitize cells to ionizing radiation. In addition, the overexpression of miR-124 would cause the modulation of EMT and stemness-related genes expression, both of which are closely related with radioresistence. The restoration of PRRX1 could rescue the effects caused by miR-124. In this research study, we have illustrated that miR-124 sensitized colorectal cancer cells to radiation treatment to some extent by downregulating PRRX1. These findings have been uncovered for the first time, thereby illustrating how miR-124 plays a key role in inducing cells resistance to ionizing radiation.

## Materials and Methods

### Patient Specimens

Clinical CRC samples were obtained from Nanfang Hospital, Southern Medical University, Guangzhou City, China. The patients had undergone maximal surgical resection followed by radiotherapy and chemotherapy. Written informed consents were obtained from the subjects who participated in this study. The tissue samples were collected and used after obtaining the approval from the Ethics Committee of Nanfang Hospital.

### Cell lines and Reagents

Human CRC cell lines, including SW480, SW620 and LOVO cells were purchased from the American Type Culture Collection (Manassas, VA, USA). HCT-116, LS-174T and HT29 were purchased from the Shanghai Cell Biology, University of the Chinese Academy of Sciences. Cells were cultured in RPMI-1640 medium (Invitrogen, Carlsbad, CA, USA) that is supplemented with 10% fetal bovine serum (Gibco, CA, USA) in a humid wet atmosphere containing 5% CO_2_ at 37°C. ANNEXIN V-FITC/7-AAD KIT was purchased from Beckman Coulter. A full-length PRRX1 cDNA that entirely lacked the 3′-UTR was purchased from GeneCopeia (Rockville, MD, USA) and subcloned into the eukaryotic expression vector pcDNA3.1 (+) (Invitrogen, USA). The pre-miR-124 sequence was amplified and cloned into pCDH-CMV-MCS-EF1-coGFP constructs (System Biosciences, California, USA). The virus particles were harvested 48 h after transfecting pCDH-CMV-miR-124 with the packaging plasmid pRSV/pREV, pCMV/pVSVG, and pMDLG/pRRE into 293T cells using Lipofectamine 2000 reagent (Invitrogen, USA). PRRX1 knockdown and control lentiviruses were purchased from GENECHEM (Shanghai, China). MiR-124 mimic, a non-specific miR control, anti-miR-124, and a non-specific anti-miR control were purchased from Thermo Scientific Dharmacon(USA).

### RNA isolation, Reverse transcription, and Quantitative real-time PCR

Total RNA was extracted from cells using TRIzol reagent (Invitrogen, USA). For miR-124, reverse transcription and qRT-PCR reactions were performed using a qSYBR-green-containing PCR kit (GenePharma, Shanghai, China). U6 snRNA was used as an endogenous control. The precursor form of miR-124 was amplified. For detecting PRRX1 mRNA, cDNA was synthesized from 1 μg of total RNA using the reverse transcription reaction kit according to the manufacturer's instructions (Promega, USA). Human GAPDH was amplified in parallel as an internal control. The primers were listed in Supplementary Table S1. All these samples were normalized to internal controls and fold changes were calculated through relative quantification (2-ΔΔCt).

### Western Blot Analysis

Equal amounts of protein were resolved by SDS-PAGE and transferred to polyvinylidene fluoride (PVDF) membrane (Millipore, USA). The membranes were blocked in 5% non-fat skim milk/TBST [20 mM Tris–HCl (pH 7.4), 150 mM NaCl, and 0.1%Tween-20] at room temperature for 1 h. Thereafter, they were detected with primary antibodies at 4°C overnight. Then, they were blotted for 1 h at room temperature with the help of an appropriate secondary antibody. Thereafter, enhanced chemiluminescence detection reagents were used (Amersham Pharmacia Biotech, USA). The primary antibody PRRX1was purchased from Abcam(UK). Caspase-3, Bcl-2, andγ-H2AX were purchased from Epitomics(UK). E-cadherin, ZO-1, Vimentin, N-cadherin were purchased from Becton, Dickinson and Company(USA). GAPDH was purchased from BOSTER(China).

### Luciferase Assay

The full-length PRRX1 3′-UTR was amplified by PCR and cloned downstream of the firefly luciferase gene in the pGL3 vector (Promega, USA). The vector was named wild-type (wt) 3′-UTR. The GeneTailor Site-Directed Mutagenesis System (Invitrogen, USA) was used to perform site-directed mutagenesis of the miR-124 binding site in PRRX1 3′-UTR: the resultant was named mutant (mt) 3′-UTR. These cells were transfected with reporter plasmids and placed in 96-well plates. After incubating the cells for 48 h, the cells were harvested and assayed using the dual-luciferase reporter assay system (Promega, Madison, WI) according to the manufacturer's instructions. Luciferase activities were normalized by β-galactosidase activity. Each experiment was performed in triplicate.

### Clonogenic Assay and Flow Cytometry

A predetermined number of cells were seeded in 6-well culture plates. Then, they were incubated for 24 h which helped in settling down these cells. The cells were treated with a range of IR doses (0, 2, 4, 6 and 8 Gy, Nasatron (Cs-137) irradiator). After incubating these cells at 37°C for 14 days, they were washed twice with PBS and stained with crystal violet solution. The number of colonies containing ≥50 cells was counted under a microscope using the following formula: Plate clone formation efficiency  =  (number of colonies/number of cells inoculated) ×100%. Survival fractions (SF) were calculated by normalisation to the plating efficiency of appropriate control groups. We used GraphPad Prism (GraphPad Software, LaJolla, CA, USA) to fit cell survival curve in accordance with a standard linear-quadratic (LQ) model. Thereafter, we obtained the values of the survival fraction of a range of IR doses. There are several important parameters in this model like SF2 (surviving fraction at 2 Gy), α (a parameter of DNA breaks caused by a shock) and β(a parameter of DNA breaks caused by two shocks).

To detect cell apoptosis, the cells were harvested and stained using 7-AAD and Annexin-V-FITC. The flow cytometry data was analyzed using BD FACS Diva software V6.1.3 (BD Biosciences).

### Animal Studies

Athymic nude mice (Guangdong Experimental Animal Center) were used for tumor implantation. These mice were about 4 to 6 weeks old. All the animal experiments strictly adhered to the Regulations for the Administration of Affairs Concerning Experimental Animals, the Chinese national guideline for animal experiment, issued in 1988. In this study, all procedures involving animals and their care were approved and performed by the Southern Medical University's Institutional Animal Care and Use Committee. The cells were harvested by trypsinization and washed twice with cold serum-free medium. Thereafter, these cells were re-suspended in 200 μl serum-free medium. For xenograft tumors assay, 5×10^6^ SW480 cells were subcutaneously injected into the back of nude mice. After tumors were detected, tumor size was measured by a slide caliper every three days. To evaluate tumor radioresistance *in vivo*, the tumors were irradiated with a single dose of 10 Gy IR 11 days after injection. Tumor volume was calculated using the formula (a×b2) ×0.5, where a and b are the long and short dimensions, respectively. Mice were killed 35 days after injection. Then, the tumors were removed. Each group contained 5 mice. These harvested tumors were imaged immediately after sacrifice.

### Statistical Analysis

All values are expressed in terms of mean values ± standard deviation. The results were analyzed using ANOVA or a two-tailed Student's t test. *P*<0.05 was considered statistically significant.

## Results

### MiR-124 Is Frequently Down-regulated in CRC Cell Lines and Tissues

**Figure 1:**
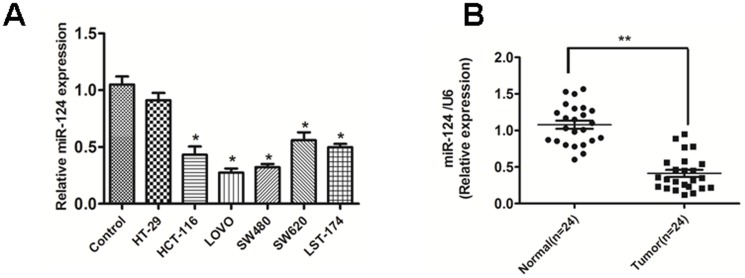
miR-124 is down-regulated both in primary CRC tissues and cell lines. (A) miR-124 was expressed at significantly lower levels in six CRC cell lines in comparison with normal colonic mucosa pooled from three healthy individuals. The figure is representative of three experiments with similar results.**P<0.01, *P<0.05. (B) The expression of miR-124 in CRC tissues and the matched normal tissues was detected by qRT-PCR and normalized to that of U6. Data are presented as individual samples (N = 24) with the line indicating the mean level.

A panel of human CRC cell lines was quantitatively analyzed to determine the expression level of miR-124. Compared with the normal colonic mucosa pooled from three healthy individuals, the expression level of miR-124 was lower in the six examined CRC cell lines. (Fig.1A)

Furthermore, we examined the expression level of miR-124 in CRC specimens and the matched normal tissues. In accordance with the data obtained from CRC cell lines, the average expression level of miR-124 was significantly lower in CRC specimenscampared to adjacent normal tissues (Fig.1B)

### MiR-124 Sensitizes Colorectal Cancer Cells to Radiation Treatment

**Figure 2:**
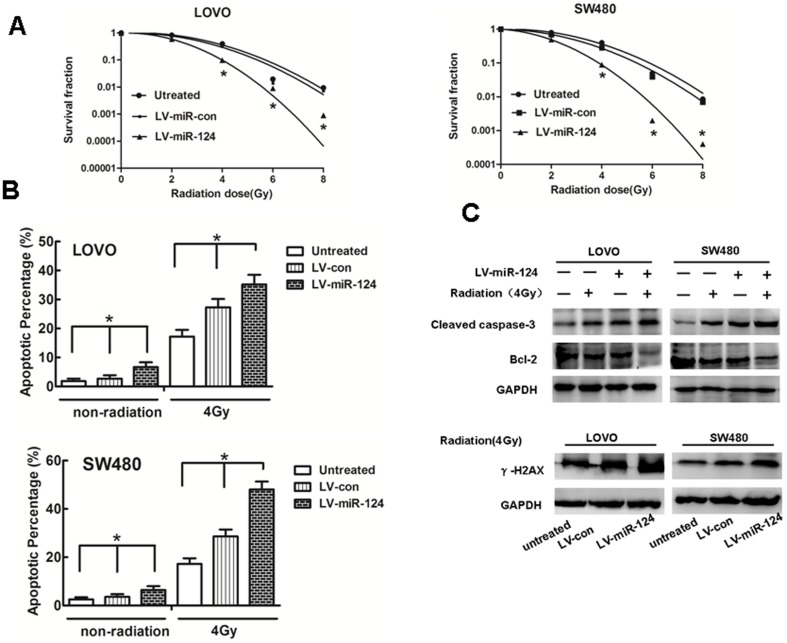
miR-124 sensitizes colorectal cancer cells to irradiation treatment in vitro. (A) LOVO and SW480 cells stably over-expression of miR-124 were treated with 0, 2, 4, 6, or 8Gy of IR. Survival fractions were calculated as described in Figure 2A. The results are presented as the means SD of values obtained in 3 independent experiments. The statistical significance of differences between the groups was calculated using Student t tests. *p<0.05. (B) Apoptosis assay showing induction of apoptosis after miR-124 over-expression, in particular combination with radiation in miR-124-overexpressed cell lines LOVO and SW480. *p<0.05. (C) Representative western blot for the effect of miR-124 over-expression or/and radiation(4Gy) on the expression of apoptosis and DNA damage related genes(Caspase-3, Bcl-2 andγ-H2AX).

The colony survival assay is considered as a canonical standard to determine radiosensitivity [29]. So, we sought to explore the effects of miR-124 on the colony survival of CRC cells in the presence of ionizing radiation. The clonogenic assay results confirmed that the overexpression of miR-124 were much more sensitive to IR than their counterparts (Fig.2A and Supplementary Table S2). It is a well-documented fact that the capacity of anti-apoptosis of cancer cells is closely associated with radioresistance. Furthermore, we measured IR-induced apoptosis in CRC cells that were transfected with miR-124 mimics or miR control. We found that when combination with IR, the overexpression of miR-124 significantly enhanced the apoptosis of cells in CRC cells than in the controls (Fig.2B). In addition, we observed that miR-124 treatment alone can decrease Bcl-2, and the effect was much stronger when combined with radiation therapy. On the other hand, caspase-3 and phosphorylation of histone H2AX (γ-H2AX) [30], an indicator of the cellular response to DNA damage increased when cells were either treated with miR-124 alone or subjected to a combined treatment of miR-124 and radiation therapy (Fig.2C). Taken together, these observations illustrated a synergistic effect between miR-124 restoration and IR.

### PRRX1 Is a Direct Target of miR-124

**Figure 3:**
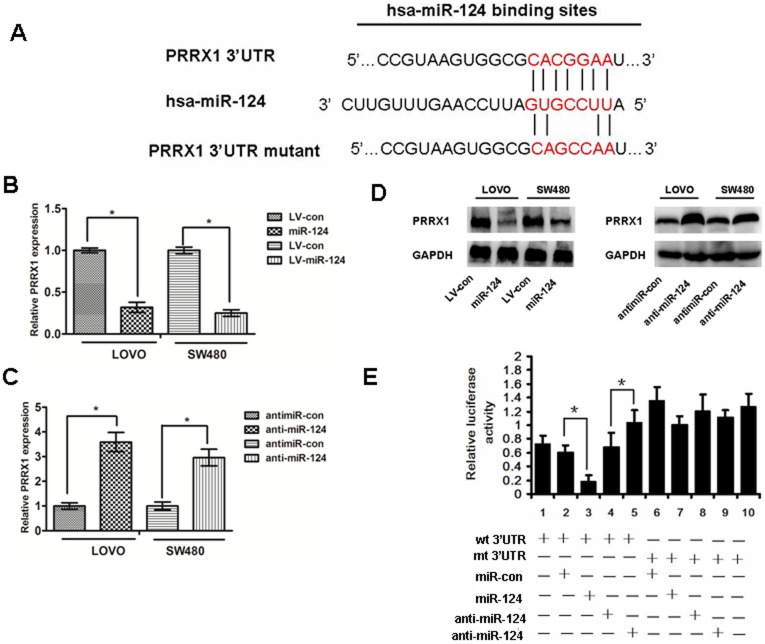
PRRX1 is a direct target of miR-124. (A) The predicted binding sequences for miR-124 within the human PRRX1 3′UTR. Seed sequences are highlighted and underlined. (B), (C) and(D) PRRX1 expression was determined in colorectal cancer cells stably overexpressed-miR-124 and cells transfected with miR-124 inhibitors, or antimiR-con by real-time PCR and Western blot analysis. ANOVA and Student t tests were used to determine the statistical significance of the differences between groups. *p<0.05. (E) Luciferase activity assays using a luciferase reporter with wild-type or mutant human PRRX1 3′UTRs were performed after co-transfection of miR-124 mimics or inhibitors into HEK293 cells. And mt 3′UTR has a significantly increase compared with wt 3′UTR.The bar graph showed the mean±SD in three independent transfection experiments. *p<0.05.

We performed a bioinformatics analysis using TargetScan and Pictar and predicted that miR-124 may target PRRX1 3′UTR region. Indeed, there was perfect base pairing between the seed sequence of mature miR-124 and the 3′UTR of PRRX1 mRNA, and these seed sequences were conserved across species (Fig.3A). To determine whether the 3′UTR of PRRX1 mRNA is a functional target of miR-214 in CRC cells, the target sequence of PRRX1 3′UTR (wt 3′UTR) or the mutant sequence (mt 3′UTR) were cloned into a luciferase reporter vector (Fig.3E). Thereafter, HEK293 cells were transfected with wt or mt 3′UTR vector and miR-124 mimics. The results showed a significant decrease of luciferase activity compared with miRNA control (Fig.3E, lanes 2 and 3; P<0.05). The activity of mt 3′UTR vector was not affected by a simultaneous transfection with miR-124 (Fig.3E, lanes 7 and 8).What's more, cotransfection with anti-miR124 and wt 3′UTR vector in HEK293 cells led to an increase of luciferase activity (Fig.3E, lanes 4 and 5; P<0.05). PRRX1 expression was detected by qRT-PCR and western blot after modulation the expression of miR-124(Fig.3B, 3C, 3D). Taken together, all these results strongly indicate that PRRX1 is a target of miR-124 in CRC cells.

### PRRX1 Knockdown Confers radiosensitivity in CRC Cell Lines

**Figure 4:**
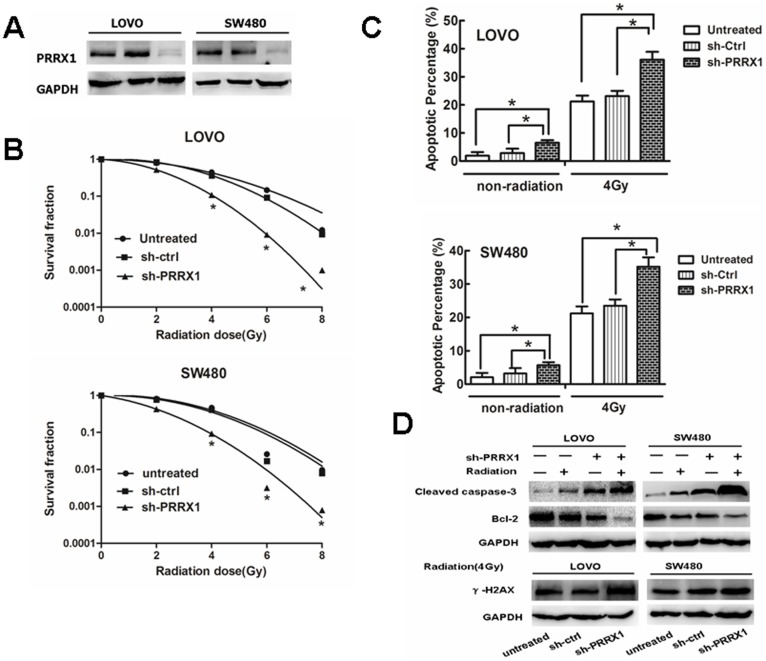
The effect of PRRX1 knockdown on radiosensitivity of CRC cells in vitro. (A) Western blot analyzed PRRX1 interference efficiency in LOVO and SW480 cells (B) The quantification of the number formed from CRC cells that were stably infected with empty vector lentiviruses (control shRNA) and PRRX1-shRNA lentiviruses (PRRX1 shRNA) or without lentiviral infection (untreated). (C) The Annexin-V assay of apoptosis for the PRRX1 knockdown cells compared with the control cells.*p<0.05. (E) Representative western blot for the effect of PRRX1 knockdown or/and radiation(4Gy) on the expressions of apoptosis related genes(Caspase-3, Bcl-2 andγ-H2AX).

Stable cell lines with shRNA -PRRX1 were established. The PRRX1 protein expression in these cells was verified by western blot analysis of the cell lysates with specific antibodies (Fig. 4A). Colony survival assay was carried out subsequently. It was found that the PRRX1-silenced, surviving fractions of cellular colonies decreased much more significantly compared with the control group when they were irradiated in different IR doses (Fig. 4B and Supplementary Table S3). Apoptosis assay showed that PRRX1 silencing combined with radiation resulted in much more apoptosis than in cases where only a single treatment was used (Fig.4C), indicating a synergistic effect. Western blot assay showed that when combined with radiation, PRRX1 silencing caused a significant down-regulation of Bcl-2 level but an up-regulation of caspase-3 expression andγ-H2AX (Fig. 4D).

### Enforced expression of PRRX1 Restores the effects of miR-124 on radiosensitivity

**Figure 5:**
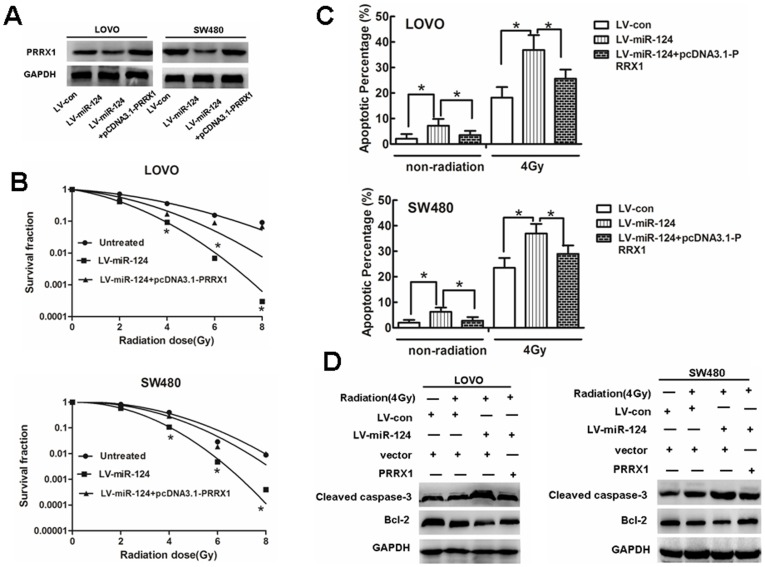
Restoration of PRRX1 expression in miR-124-overexpressed cells rescues the effects of miR-124 on radiosensitivity. (A) PRRX1 expression was detected by western blot after transfecting pcDNA3.1-PRRX1 into miR-124-overexpressed cells. (B) Cells were followed by treatment as described in Figure 2A. Survival fractions were calculated for each group. The results are presented as the means ±SD of values obtained in 3 independent experiments. ANOVA or Student t tests were used to determine the statistical significance of the differences between groups. Statistical significance(*P<0.05) is indicated vs LV-con and miR-124 group. (C) Representative western blot for the effect of PRRX1 restoration or/and radiation(4Gy) on the expressions of apoptosis related genes(Caspase-3, Bcl-2 andγ-H2AX).

To elucidate whether the effect of miR-124 on radiosensitivity was mediated by repression of PRRX1, pcDNA3.1-PRRX1 was transfected into miR-124-overexpressed cells. PRRX1 expression was verified by western blot (Fig.5A). The results showed that PRRX1could rescue the effects of miR-124. Clonogenic assay and cell apoptosis suggested that the ectopic expression of PRRX1 significantly reduced miR-124-induced radiosensitivity (Fig.5B and Supplementary TableS4, Figure.5C). Western blot analysis also indicated PRRX1 could restore the expression of caspase-3 and Bcl-2, which was triggered by miR-124 (Fig.5D). These observations indicated that miR-124 sensitized cells to IR by downregulating the expression of PRRX1.

### Ectopic PRRX1 Reverses the expression of EMT and Stemness-related genes in Stably miR-124-overexpressed cell Lines

**Figure 6:**
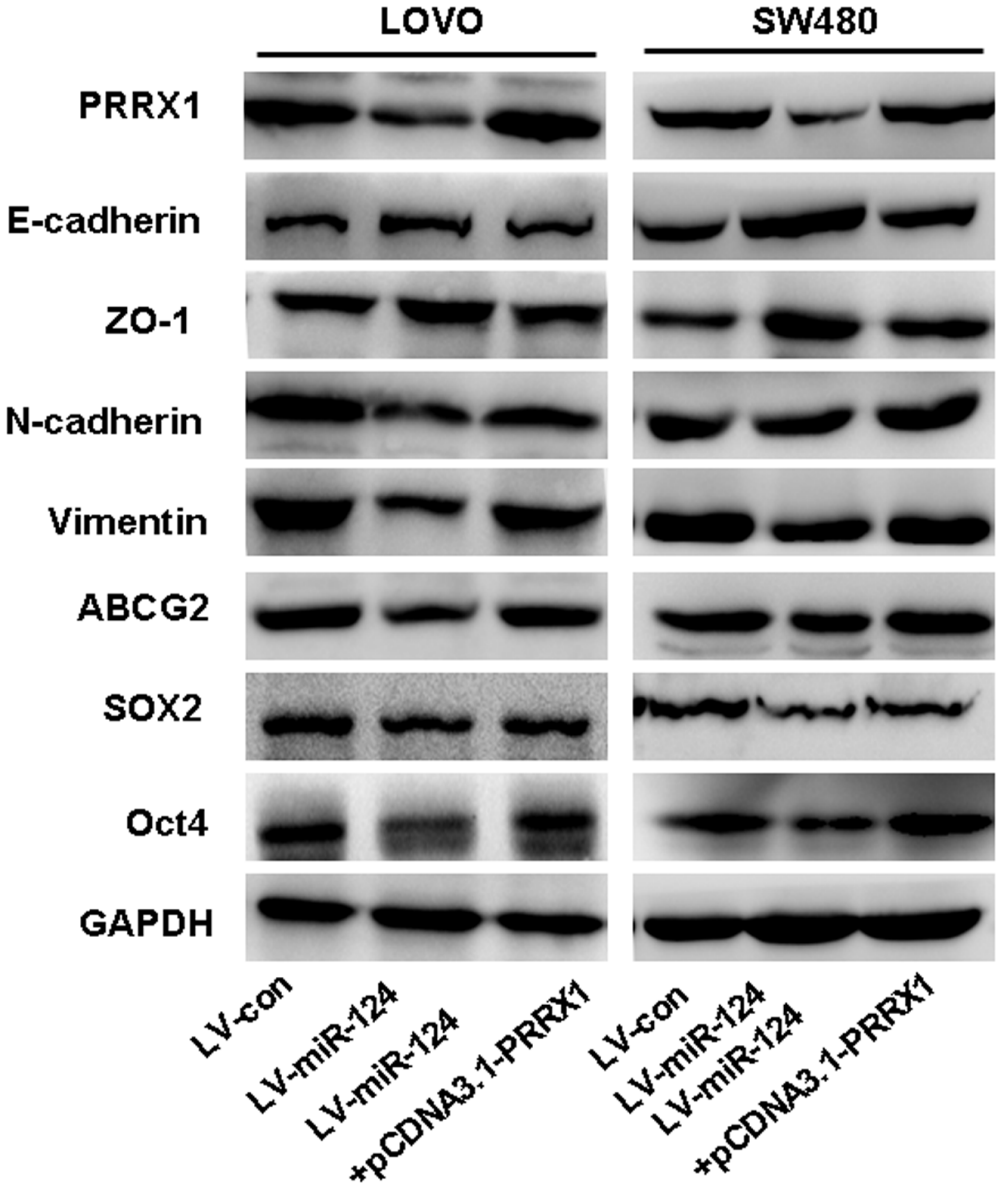
Detecting EMT and stemness-related genes expression by western blot. Western blot analyzed the EMT-related genes like E-cadherin, ZO-1, Vimentin and N-caherin and stemness-related genes such as ABCG2, SOX2 and Oct4 in miR-124-tranfected cells and miR-124-PRRX1 co-transfected cells compared with control group. Data suggested over-expression of miR-124 could down-regulate Vimentin, N-cadherin, ABCG2, SOX2 and Oct4 expression and up-regulate E-cadherin, ZO-1 expression, while over-expression of PRRX1 could rescue this effect.

In recent research studies, it was found that PRRX1 induces EMT and promotes the stemness phenotype in CRC cell lines [27]. In recent research studies, it has been reported that EMT is associated with cancer stem cells. Furthermore, the loss of E-cadherin and the subsequent EMT promoted radioresistance in human tumor cells [31]–[33]. Recent studies have linked the CSC phenotype to tumor cells undergoing EMT, which illustrates the complex relationship between EMT and CSCs [34]–[40]. We wonder whether miR-124 brings about this effect by downregulating PRRX1.Thereafter, we transfected pcDNA3.1-PRRX1 into miR-124-overexpressed CRC cell lines SW480 and LOVO. Western blot analysis showed that PRRX1 could reverse the expression of EMT and stemness-related genes caused by overexpression of miR-124 (Fig.6).

### In vivo Tumor Xenograft Radiosensitivity Assay

**Figure 7:**
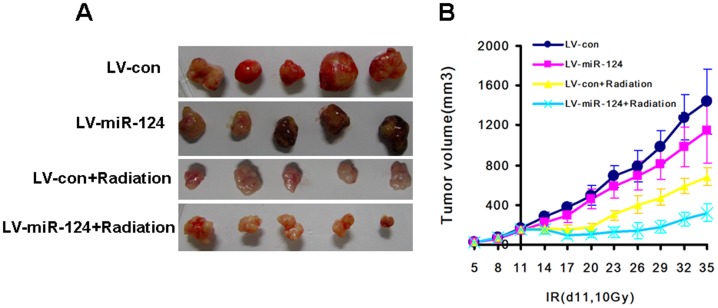
miR-124 sensitizes colorectal cancer cells SW480 to irradiation treatment in vivo. (A) Over-expression of miR-124 sensitized SW480 tumor xenografts to IR in vivo. Tumor sizes were measured with caliper and irradiation was delivered to tumors area on the eleventh day(IR:10Gy, d11). (B)The volume curves of xenografts generated by LV-con, LV-miR-124, LV-con+Radiation, LV-miR-124+Radiation. (*p*<0.05).

To determine whether miR-124 sensitizes tumors to IR *in vivo*, we irradiated the tumor area just once using a dose of 10 Gy 11 days after injection. Despite receiving the same dose of radiation (d11,10Gy) (Fig.7A,7B), the size of xenografts derived from miR-124-overexpressed cells were much smaller than that derived from control treated cells. These results indicated that miR-124 caused an *in vivo* sensitization of tumors to radiation.

## Discussion

In CRC clinical management, an acquired and intrinsic radioresistance is a challenging obstacle. We need to conduct more research studies to address this challenging problem. The acquisition of radioresistance is a complicated process, involving the overexpression of DNA repair proteins [41], [42], aberrant activation of multiple signalling pathways [43]–[45], angiogenesis [46], [47], cancer stem cells [48], and autophagy [49], [50]. Several previous research studies have shown that miRNAs were closely related to tumor radiosensitivity. This is because miRNAs have the ability to increase and decrease radiosensitivity of tumors [8], [23], [51]–[54]. Given that miRNAs have the ability to regulate multiple oncogenic processes such as responsiveness to therapy, we must explore the role of miRNAs in radiation resistance. Resistance to IR has contributed to treatment challenges of patients suffered from CRC. Thus, understanding the molecular mechanisms underlying radiation sensitivity and resistance remains an important pursuit.

In this study, we found that miR-124 was downregulated in both CRC-derived cell lines and clinical CRC samples compared with normal tissues. To gain an insight into the function of miR-124, we performed *in vitro* experiments and human xenograft studies. These research studies have illustrated that overexpression of miR-124 could radiosensitize CRC cells and miR-124 knockdown induced cell resistance to irradiation.

We identified PRRX1 was a direct target of miR-124 by luciferase assay. To further reveal the functions of PRRX1 on cell radiosensitivity, we constructed stable PRRX1-knockdown cell lines LOVO and SW480 and found that PRRX1 knockdown induced cell sensitivity to irradiation in a manner that is similar to the effect induced by the overexpression of miR-124. Moreover, PRRX1 up-regulation rescued the effects of miR-124-overexpression on radiosensitivity of cells. These results indicate that the effect of miR-124 on cell sensitivity to irradiation is partly mediated by repressing the expression of PRRX1. As reported previously, PRRX1 induced EMT and enhanced self-renew properties. Our results suggest that the up-regulation of miR-124 increases the expression of epithelial markers like E-cadherin and ZO-1 while simultaneously decreasing the expression of mesenchymal markers such as N-cadherin and Vimentin. Furthermore, the up-regulation of miR-124 led to a simultaneous downregulation in the expression of stemness-related genes, namely, ABCG2, SOX2, and Oct4. In addition, the overexpression of PRRX1 could rescue the effect of miR-124 on EMT by stemming genetic alterations. In recent times, it has been reported that EMT was associated with cancer stem cells. Moreover, cells undergoing EMT showed greater radioresistance in human tumor cells [26], [31]–[33] Taking these observations into consideration, we inferred that miR-124 could radiosensitize CRC cells by downregulating PRRX1, which is associated with EMT and cancer stem cells. However, all these observations need to be further investigated and verified through more research work. We investigated the role of miR-124 in regulating radiosensitivity, which may significantly affect cancer biology and cancer therapy. Based on these observations, we hypothesized that the downregulation of PRRX1 reversed EMT and simultaneously weakened the self-renewal properties of cells, both of which are closely related to radioresistence.

In conclusion, we provide evidence that miR-124 sensitizes CRC cells to radiation treatment by inhibiting PRRX1. This indicates that miR-124 is an attractive prognostic/predictive biomarker, which can be used in diagnosing CRC cases. Moreover, we have developed a new approach to sensitizing radioresistant cancers by targeting miR-124.

## Supporting Information

Table S1 **Primers for miR-124 and PRRX1 quantification.**
(DOC)

Table S2 **Radiosensitivity parameters after overexpression of miR-124.**
(DOC)

Table S3 **Radiosensitivity parameters after PRRX1 knockdown.**
(DOC)

Table S4 **Radiosensitivity parameters after overexpression of PRRX1 in miR-124-overexpressed cell lines.**
(DOC)
