# Supplementary material for: MiR-124 Radiosensitizes Human Colorectal Cancer Cells by Targeting PRRX1
Source: PLoS One. 2014 Apr 4;9(4):e93917. doi: 10.1371/journal.pone.0093917 (PMC3976353; doi:10.1371/journal.pone.0093917)
Supplement: Table S1 — Primers for miR-124 and PRRX1 quantification. (DOC) [file pone.0093917.s001.doc]

**Table S1. Primers for miR-124 and PRRX1 quantification.**

| Name | Sequence(5’-3’) | Tm(°C) | Amplicon (bp) |
| --- | --- | --- | --- |
| miR-124-RT | GTCGTATCCAGTGCAGGGTCCGAGGTATTCGCACTGGATACGACGGCATTCT | 87.8 |  |
| miR-124-F | GATACTCATAAGGCACGCGG | 60.6 | 64 |
| miR-124-R | GTGCAGGGTCCGAGGT | 57.9 |  |
| PRRX1-F | CAGGCGGATGAGAACGTGG | 62.7 | 213 |
| PRRX1-R | AAAAGCATCAGGATAGTGTGTCC | 60.3 |  |
| U6-F | CGCTTCGGCAGCACATATAC | 59.4 | 60 |
| U6-R | CAGGGGCCATGCTAATCTT | 57.5 |  |
| GAPDH-F | GGAGCGAGATCCCTCCAAAAT | 61.6 | 197 |
| GAPDH-R | GGCTGTTGTCATACTTCTCATGG | 60.9 |  |
