# Supplementary material for: MiR-124 Radiosensitizes Human Colorectal Cancer Cells by Targeting PRRX1
Source: PLoS One. 2014 Apr 4;9(4):e93917. doi: 10.1371/journal.pone.0093917 (PMC3976353; doi:10.1371/journal.pone.0093917)
Supplement: Table S2 — Radiosensitivity parameters after overexpression of miR-124. (DOC) [file pone.0093917.s002.doc]

**Table S2. Radiosensitivity parameters after overexpression of miR-124.**

| **Cell** | **Group** | **SF2** | **α** | **β** |
| --- | --- | --- | --- | --- |
| **LOVO**  ***P* Value** | LV-con | 0.76±0.018 | 0.046±0.0035 | 0.088±0.0034 |
|  | LV-miR-124 | 0.498±0.008 | 0.068±0.0039 | 0.156±0.0058 |
|  |  | ＜0.01 | ＜0.05 | ＜0.05 |
| **SW480**  ***P* Value** | LV-con | 0.69＜0.001±0.016 | 0.033±0.0012 | 0.073±0.0031 |
|  | LV-miR-124 | 0.48±0.0102 | 0.11±0.0089 | 0.125±0.0075 |
|  |  | ＜0.01 | ＜0.05 | ＜0.05 |

*(SF2, surviving fraction at 2 Gy; α, Parameter of DNA breaks caused by a shock; β, Parameters of DNA breaks caused by two shocks;)*
