# Supplementary material for: MiR-124 Radiosensitizes Human Colorectal Cancer Cells by Targeting PRRX1
Source: PLoS One. 2014 Apr 4;9(4):e93917. doi: 10.1371/journal.pone.0093917 (PMC3976353; doi:10.1371/journal.pone.0093917)
Supplement: Table S3 — Radiosensitivity parameters after PRRX1 knockdown. (DOC) [file pone.0093917.s003.doc]

**Table S3. Radiosensitivity parameters after PRRX1 knockdown.**

| **Cell** | **Group** | **SF2** | **α** | **β** |
| --- | --- | --- | --- | --- |
| **LOVO**  ***P* Value** | sh-ctrl | 0.79±0.089 | 0.074±0.0056 | 0.070±0.0012 |
|  | sh-PRRX1 | 0.52±0.034 | 0.152±0.0082 | 0.112±0.0065 |
|  |  | ＜0.01 | ＜0.05 | ＜0.05 |
| **SW480**  ***P* Value** | sh-ctrl | 0.69±0.078 | 0.059±0.015 | 0.077±0.0023 |
|  | sh-PRRX1 | 0.43±0.023 | 0.248±0.087 | 0.097±0.0045 |
|  |  | ＜0.01 | ＜0.05 | ＜0.05 |

*(SF2,surviving fraction at 2 Gy; α, Parameter of DNA breaks caused by a shock; β, Parameter of DNA breaks caused by two shocks;)*
