# Supplementary material for: MiR-124 Radiosensitizes Human Colorectal Cancer Cells by Targeting PRRX1
Source: PLoS One. 2014 Apr 4;9(4):e93917. doi: 10.1371/journal.pone.0093917 (PMC3976353; doi:10.1371/journal.pone.0093917)
Supplement: Table S4 — Radiosensitivity parameters after overexpression of PRRX1 in miR-124-overexpressed cell lines. (DOC) [file pone.0093917.s004.doc]

**Table S4. Radiosensitivity parameters after overexpression of PRRX1 in miR-124-overexpressed cell lines.**

| **Cell** | **Group** | **SF2** | **α** | **β** |
| --- | --- | --- | --- | --- |
| **LOVO**  ***P* Value** | LV-miR-124 | 0.41±0.052 | 0.273±0.059 | 0.081±0.008 |
|  | LV-miR-124+pcDNA3.1-PRRX1 | 0.59±0.0415 | 0.170±0.063 | 0.055±0.006 |
|  |  | ＜0.05 | ＜0.05 | ＜0.05 |
| **SW480** | LV-miR-124 | 0.58±0.049 | 0.353±0.022 | 0.093±0.004 |
| ***P* Value** | LV-miR-124+pcDNA3.1-PRRX1 | 0.76±0.096 | 0.270±0.013 | 0.065±0.003 |
|  |  | ＜0.05 | ＜0.05 | ＜0.05 |

*(SF2,surviving fraction at 2 Gy; α, Parameter of DNA breaks caused by a shock; β, Parameter of DNA breaks caused by two shocks;)*
